# Genotypic Variability in Architectural Development of Mungbean (*Vigna radiata* L.) Root Systems and Physiological Relationships With Shoot Growth Dynamics

**DOI:** 10.3389/fpls.2021.725915

**Published:** 2021-08-19

**Authors:** Vijaya Singh, Michael Bell

**Affiliations:** ^1^Queensland Alliance for Agriculture and Food Innovation, The University of Queensland, St Lucia, QLD, Australia; ^2^The School of Agriculture and Food Sciences, The University of Queensland, Gatton, QLD, Australia

**Keywords:** intact root growth, root system architecture, rooting depth, phosphorus acquisition, maturity type

## Abstract

Selection for root system architectures (RSA) to match target growing environments can improve yields through better adaptation to water and nutrient-limiting conditions in grain legume crops such as mungbean. In this study, the architectural development of root systems in four contrasting mungbean varieties was studied over time to explore their relationships to above-ground growth and development. Key findings suggested that early maturing mungbean varieties were characterized by more rapid root elongation rates and leaf area development, resulting in more vigorous root and shoot growth during early growth stages compared with a late maturing variety. The early maturing varieties also showed root morphological traits generally adapted to water-limited environments, such as deeper, longer and lighter roots. Early maturing varieties more rapidly colonized the top 10–20 cm of the soil profile during early growth stages, whereas the later maturing variety developed less prolific but 20–50% thicker roots in the same profile layers in later stages of crop growth. The diversity of root characteristics identified in these commercial varieties suggests that there are opportunities to combine desirable root traits with maturity types to target different production environments. Examples include deeper, longer, and thinner roots for crops to exploit deep profile reserves of water and nutrients, and thicker and shallower root systems for crops grown in shallow soils with stratified nutrient reserves and/or more favorable in-season rainfall.

## Introduction

Mungbean (*Vigna radiata* [L.] Wilczek) is an economically important tropical grain legume crop that has the potential to play a key role in managing soil fertility as a nitrogen-fixing legume in crop rotation systems (Araujo et al., 2015; Foyer et al., 2016; Singh et al., 2016a,b). There is increasing interest in growing a higher frequency of grain legume crops in broadacre grain cropping systems, especially mungbean and chickpea (*Cicer arietinum*), due to strong market demand and high commodity prices. In addition to these economic incentives, grain legumes can also deliver multiple benefits through a smaller environmental footprint, improved stock and human health, reduced use of synthetic nitrogen (N) due to biological N fixation, reduced soil pathogen populations and provide an option to increase plant-based dietary intake of minerals, vitamins and fiber (Parida and Das, 2005; Arnoldi et al., 2014; Vaz Patto et al., 2014). However, the reliability of mungbean production and its profitability in crop rotations needs to be improved if the strong market demand for mungbean is to be met. There are numerous challenges to reliable mungbean production in the major growing regions of India and Australia, especially abiotic factors in interaction with changing climatic conditions (Beebe et al., 2011; Basu et al., 2016; Singh et al., 2016a). Limited availability of water and poor soil fertility are widespread, resulting in poor crop growth and unprofitable mungbean yields (Araujo et al., 2015).

Plant growth and development are dependent on root morphology and root system architecture (RSA) that facilitate the acquisition of water and nutrients. Under adverse soil or environmental conditions, RSA could be a critical factor in determining profitable crop production (Lynch, 1995; de Dorlodot et al., 2007; Ye et al., 2018). The spatial distribution of roots in soil can influence the extent and timing of access to water and nutrients, thus impacting yield potential (Lynch, 1995; de Dorlodot et al., 2007; Hammer et al., 2009; Liang et al., 2017; Ye et al., 2018). Modifications to root systems may therefore improve adaptation to water- and nutrient-limiting conditions that are constraining yields in mungbean crops. However, genetic improvement of crop root systems requires knowledge of the intra-species variability in key root parameters and RSA and how these are controlled genetically (O'Toole and Bland, 1987). An understanding of the relationships between RSA and plant productivity is also necessary before effective breeding and management strategies can be developed (Gowda et al., 2011; Henry et al., 2011; Joshi et al., 2017). Despite its importance, few studies have explored the potential for including RSA as a selection strategy in crop improvement programs. Lawn and Rebetzke (2006) identified substantial variation for traits of potential agronomic, adaptive or taxonomic interest among 115 accessions of mungbean, mainly from Australia, West Timor, Papua New Guinea and India. However, genetic variation in RSA and relationships with plant growth and development in mungbean are largely unknown (Pratap et al., 2013, 2014; Singh et al., 2016b).

While RSA plays an important role in water and nutrient acquisition and plant growth, most studies that have characterized root architectural parameters represent a snapshot at a specific time (Manschadi et al., 2008; Singh et al., 2012, 2016b; Uga et al., 2013). Observations made at maturity cannot fully explain the relationship between RSA and plant growth and yield accumulation, as root systems are known to be plastic in nature and can interact dynamically with soil physical, chemical and biological factors at different stages of a growing season (Lynch, 1995; Wu et al., 2016; Chen et al., 2017). Additionally, most studies have used destructive techniques such as soil coring or trench wall methods to describe the RSA at the end of the experiment. These techniques are time consuming and tedious and cannot quantify the intact root growth patterns in terms of rate of growth, branching, spatial distribution and occupancy of the soil volume over time (McCully, 1995; Chen et al., 2017). Development of roots occurs in synchrony with shoot growth (Wang et al., 2006), so characterization of RSA over time is important to understand the interactions between RSA and shoot growth dynamics. The impact of differences in RSA established during vegetative growth will be maintained during reproductive growth in determinate species. However, in semi-determinate to indeterminate species like mungbean and other tropical/subtropical grain legumes, root growth occurs during both vegetative and reproductive phases, and so measurement of RSA at the end of an experiment cannot identify when RSA differences were likely to be affecting the critical growth stages of the plant. Multiple destructive samplings at critical growth stages are therefore needed to study the dynamic nature of root growth patterns relative to shoot growth and yield parameters.

Field excavation, trenches and soil coring have been used to quantify root growth and RSA in field studies (Trachsel et al., 2011; Vansteenkiste et al., 2014), but these methods are labor intensive and information on the actual root architecture is often lost. Recent advancements in the technologies used to measure static and dynamic root growth have included non-invasive methods where plants are grown in artificial gel media (Manschadi et al., 2008; Hargreaves et al., 2009), CT scans/X-ray micro-tomography (Hochholdinger, 2009; Mooney et al., 2012; Mairhofer et al., 2013) and MRI of intact soil cores (Schulz et al., 2013).

However, these techniques are only successful for very young plants (a few days to a couple of weeks old) growing in controlled conditions and exhibiting simple branching patterns. Minirhizotrons have also been used to non-destructively measure root growth, with this technique permitting tracing or imaging of intact roots on a transparent surface of a growth chamber (Singh et al., 2012; Downie et al., 2015). However, despite the confined rooting volumes used in such systems, roots seen on the transparent surface typically represent only *ca*. 20% of the total roots of a plant. Tracing and analysis of root images collected over time from these systems is very time consuming and there is often not much success in differentiating contrasting root systems (Singh et al., 2010, 2011, 2012).

The objectives of this study were therefore to develop a technique to study the intact root system of mungbean plants as they grow, using four contrasting mungbean varieties to characterize the morphological and architectural development of intact root systems. The novelty of this study was to understand the relationships between growth of the entire root system with the growth and development of the above-ground plant components during a growing season. This contrasts with most studies that explore these relationships at a single time point at the end of an experiment or growing season. A subsequent study uses these same varieties to explore responses to different phosphorus fertilizer application strategies in terms of RSA, plant growth and nutrient acquisition.

## Materials and Methods

### Plant Material

The experiment compared four commercial mungbean varieties from Australia, with contrasting growth characteristics and maturity classes. All varieties were produced by the National Mungbean Improvement Program, Queensland Department of Agriculture and Forestry (DAF), Australia:

Jade-AU (3511-9 × VC 2768A, released in 2013)—a mainstay variety for Australia, producing large shiny seeds; a variety that retains green leaf area until harvest, and so can respond to residual soil water and nitrogen.

Berken (released 1975, direct introduction from the USA, no pedigree history)—older variety with low yield potential and highly susceptible to plant diseases; a more determinate growth pattern that is characterized by canopy senescence during pod filling and at grain maturity.

Celera II-AU (M 773 × OAEM58-62, released 2015)—a small seeded, short statured variety with distinguishing leaf morphology; resistant to the bacterial disease halo blight, caused by *Pseudomonas savastanoi* pv. *Phaseolicola*.

Putland (Berken × CPI20141, released 1991)—a small-seeded, photoperiod sensitive variety that produces large biomass.

The varieties Jade, Berken and Celera II are classified as early maturing (50–60 days), while the variety Putland is characterized by a much longer growing season (75–85 days) that is influenced by photoperiod. Within the early maturing varieties, Berken is slightly earlier than the Celera II (Lawn, 1979).

### Experimental Site and Unit

**Figure 1:**
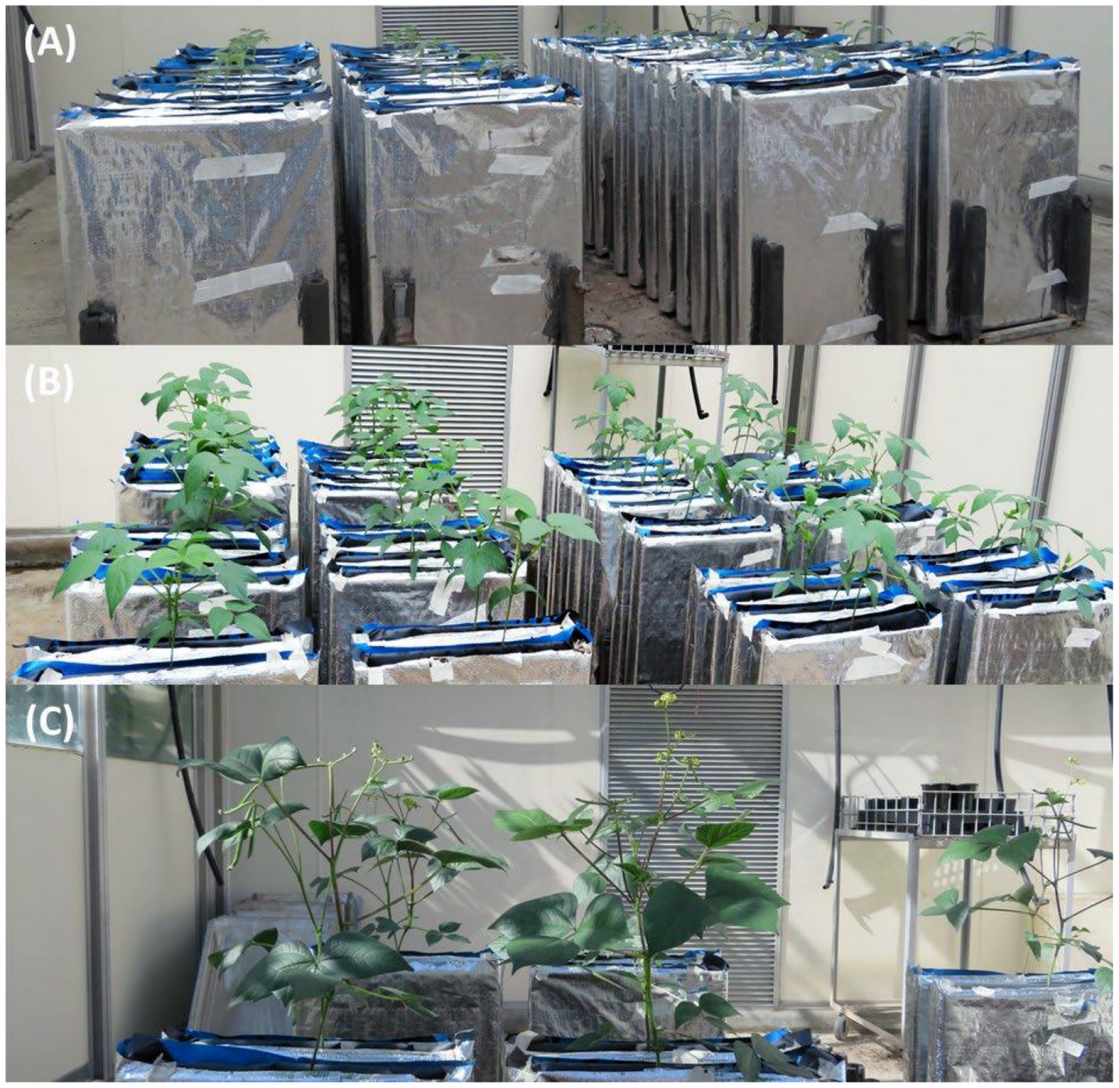
Mungbean plants growing in root chambers in a temperature-controlled glasshouse with four replications. **(A)** Before harvest 1, **(B)** harvest 2, and **(C)** harvest 3.

The experiment was conducted in a temperature-controlled glasshouse at The University of Queensland, Brisbane, Australia (27°23′S, 153°06′E). Purpose-built root observation chambers were constructed from perspex sheets, with chamber dimensions being 60 cm high, 40 cm wide and 3 cm thick. Transparent perspex (8 mm thick) sides were used to enable viewing and scanning of roots. The perspex sheets were screwed to the metal frame of the chamber and the back of the chambers was lined with black plastic to allow easy removal at harvest. The chambers were wrapped in silver insulation to prevent exposure of the roots to light and to minimize fluctuations in soil temperature (Figure 1).

Each chamber was filled with 9 kg air dried soil (Vertosol—Isbell, 1986), which was collected in bulk from the top 15 cm layer of the soil profile from research fields on the Gatton Campus (Queensland, Australia). This soil was characterized by a clay content of 35–40%, pH_CaCl2_ 8.0, electrical conductivity_1:5soil−water_ 0.4 dS/m, 24 mg Cl/kg soil, organic carbon 0.6%, Nitrate-N 79 mg/kg soil, Bicarbonate extractable (Colwell) P 18 mg/kg soil and a Cation Exchange Capacity of 51.7 cmol(+)/kg with 8.8% exchangeable sodium. Soil was not assessed as being deficient in any macro or micronutrients. The soil was air dried in the sun and then crushed to 5 mm size with a jaw crusher before being thoroughly mixed to provide a homogenous growing medium. The bulk density of the packed soil in the root observation chamber was estimated to be approximately 1.25 Mg/m^3^. A complete liquid fertilizer (Peters Professional Water-Soluble Fertilizer Hydro-sol, ScottsSierra Horticultural Products Co., Marysville, OH, USA) was added to the soil before planting to ensure nutrients were non-limiting.

### Experimental Design

The treatments consisted of four varieties sampled at each of four harvest dates spaced 10 days apart commencing 20 days after emergence and designed to cover key vegetative and reproductive growth stages. Four replicate root chambers of each combination of variety and harvest date were laid out in a split plot design with harvest dates deployed as the main plots and varieties as the subplots (Figure 1).

### Growing Conditions

Soil in each chamber was saturated and drained to reach field capacity before sowing, with the wetting up process typically taking 2–3 days. Once drained, three seeds of one of the four contrasting varieties were sown in each chamber and gradually thinned to one established plant 4 days after germination. The chambers were arranged on a stand that gave a plant-to-plant spacing of 20 cm, and chambers were watered from the top every 10 days to return the soil to field capacity and avoid development of water stress.

### Measurements

Before each harvest, plant height (from base to top of the stem) and total number of branches (at the nodes) were recorded. Plants were then destructively sampled by collecting the shoot of each plant above the base of the stem and separating into stem, leaf and pod fractions (pods were present in the last two harvests of the early maturing varieties only). The total number of leaves, number of fully expanded leaves, and leaf area (using a LICOR Planimeter (Li-3000 leaf area meter) were recorded.

**Figure 2:**
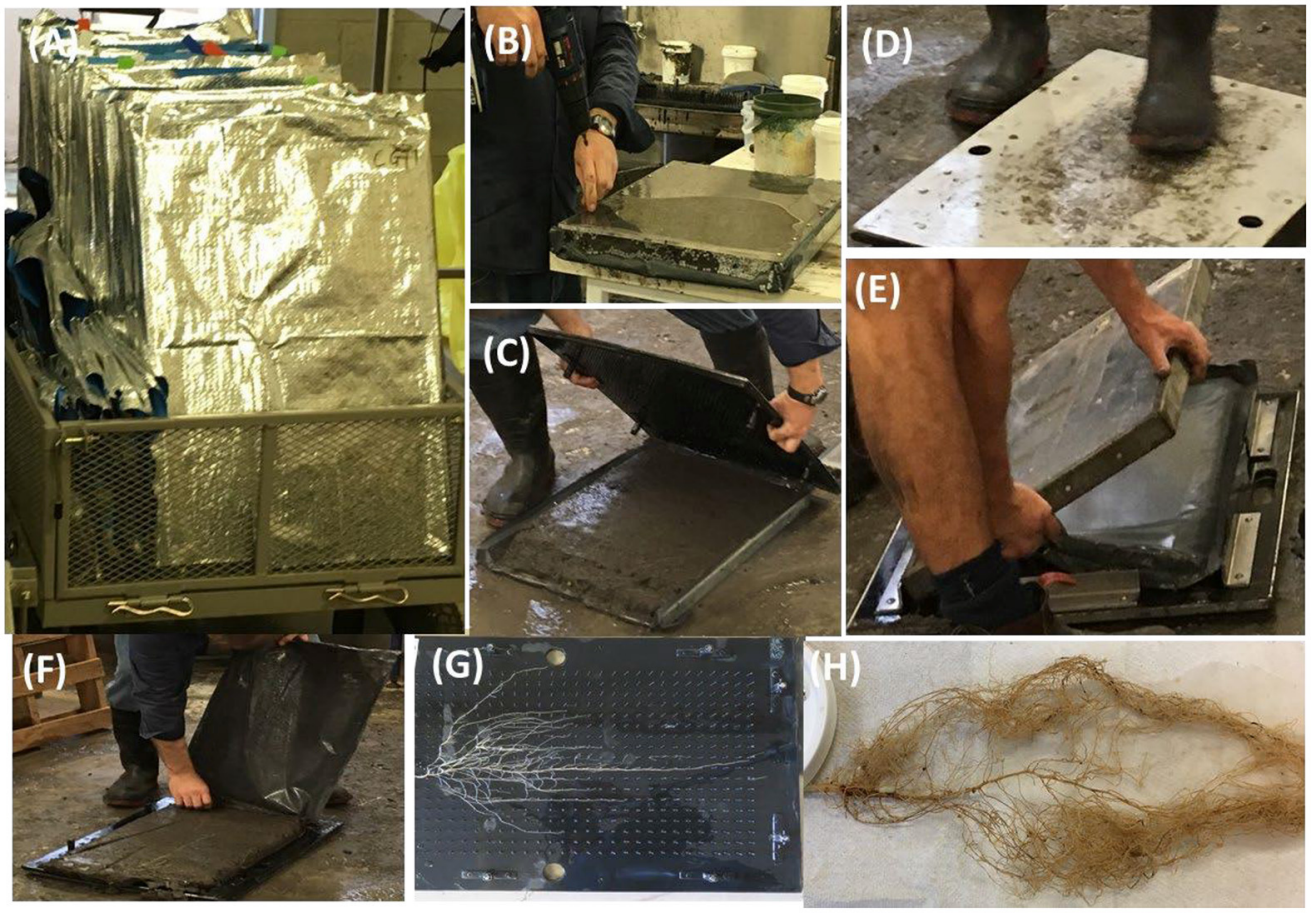
Recovering intact root system architecture. **(A)** Root chambers brought in the soil laboratory, **(B)** screws are removed from the perplex panels, **(C)** top panel is removed so that pin board can be inserted, **(D)** pinboard is inserted with pressure, **(E)** panel of other side is removed, **(F)** black plastic holding the soil is removed, **(G)** a washed intact root system on the pinboard, and **(H)** a washed root system after storing in the ethanol for further analysis.

After removing the shoot, chambers were saturated with water overnight, after which each chamber was laid flat and the top perspex plate was removed. Purpose-built plywood pinboards that matched the chamber dimensions and fitted with 3 cm long black nails positioned in a 2 × 2 cm grid were then placed on top of the exposed soil (Figure 2). The moist soil allowed the nails to be easily pushed into the soil of the chamber, while preserving most of the intact root system architecture. The pinboards plus soil and roots were then held erect while the soil was washed from the pinboard using a very fine, low pressure water spray to minimize disturbance of the intact root system (Figure 2). The total number of nodules on the root system was recorded, the length of the tap root was manually measured with a ruler and the diameter of the tap root 1 cm below the soil surface was measured with a digital caliper. The washed root system was then imaged with a digital camera (Canon, SX720 HS) mounted on a tripod and the images were converted to high-contrast black and white images using Adobe Photoshop software. Images were initially cropped to the same size and then image adjustment and threshold tools were used to convert the image into black and white. The average root angle of the first and second lateral branches was determined using “openGelPhoto.tcl” (www.activestate.com/activetcl), free software that calculates the angle of individual roots relative to the vertical plant (for example, Joshi et al., 2017). After imaging, the roots were stored in 70% ethanol in a cold room (4°C) for later manual measurements of the tap root length, number of nodules and total root dry weights.

Dry matter of each plant part (stem, leaf, pods and roots) was obtained after drying in a dehydrator for four days at 70°C. Development of leaf area, root surface area, top mass (shoot + pods), shoot mass (leaf + stem) and root mass were quantitatively determined for each growth stage, and these data were also expressed as values relative to the maximum value recorded for each parameter during the experiment. While this was typically the last harvest (H4) for parameters relating to mass, other parameters sometimes achieved their maxima earlier in the experiment (e.g., H3 for leaf area). The relative values were primarily used to contrast patterns of above and below ground growth and development for a variety, and between varieties.

### Intact Root Characteristics

Images of intact roots were analyzed using WinRhizo™ Pro 2019 software (https://regent.qc.ca/assets/winrhizo_software.html). The image was acquired from the camera by electing the “origin” setting in the software, and the following settings were chosen—image resolution was 600 dpi, the root background was changed to gray scale and calibration was performed by marking the length and width of the image (60 × 40 cm). The saved calibration was loaded onto each image before the start of the analysis. Before loading the calibration, the root diameter classes were changed into 10 different widths with a width interval of 0.25 mm. The total root length (cm), total root surface area (cm^2^), root surface area in top and bottom 30 cm of the chambers, mean root diameter (mm) and the number of root tips, forks and crossings were determined from image analysis. The specific root mass (g/cm) was determined from total root weight divided by total root length. Images of intact roots were also analyzed based on vertical distribution within the root chamber, with the top 30 cm and bottom 30 cm analyzed separately for later growth stages (i.e., H3 and H4) when roots had started to reach the bottom of the root chambers. These analyses were conducted to determine whether varieties differed in RSA of the shallow (top 0–30 cm) and deeper (30–60cm) parts of the soil profile.

### Statistical Analysis

Analysis of variance was performed using Genstat windows 18th edition (VSN International, 2015). A split plot treatment structure was used in the analysis, with harvest times as the main plots and varieties as the subplots. Least significant differences of means at 5% probability were used to compare differences between varieties for the various parameters.

## Results

An analysis of variance table showing the significance of main effects (varieties and harvest) and their interactions on selected plant parameters is presented in Table 1, with a more complete presentation of a wider set of parameters provided in Supplementary Table 1 (ST1). Varietal differences were highly significant (*P* < 0.001) for the majority of all parameters. However, there were also many highly significant interactions (*P* < 0.001) between varieties and time of harvest, indicating rates of growth and development varied significantly between varieties as the development of the plant progressed from early growth toward pod formation and grain filling. These interactions were explored in detail for above ground biomass and leaf area accumulation (Table 1), and subsequently in an examination of the relationship between above and below ground growth and development.

**Table 1:** Leaf area development and accumulation of above and below ground biomass in four mungbean varieties with contrasting maturities.

**Harvests**	**Varieties**	**Leaf area (cm^**2**^)**	**Shoot dry weight (g)**	**Pod dry weight (g)**	**Root dry weight (g)**	**Root: shoot ratio**
Harvest 1	Jade	37.5a	0.14a	–	0.07a	0.51f
	Putland	20.0a	0.09a	–	0.02a	0.20ab
	Berken	39.3a	0.18a	–	0.07a	0.4de
	Celera II	33.3a	0.12a	–	0.042a	0.36d
Harvest 2	Jade	251.4c	1.12bc	–	0.39b	0.34d
	Putland	175.4b	0.67b	–	0.25b	0.38d
	Berken	259.4c	1.05bc	–	0.35b	0.34d
	Celera II	210.8b	0.83bc	–	0.28b	0.34d
Harvest 3	Jade	489.9e	5.59	–	1.06c	0.19a
	Putland	642.4g	4.67de	–	1.17c	0.25bc
	Berken	425d	5.26f	–	0.84c	0.16a
	Celera II	608.5g	5.14f	–	0.91c	0.18a
Harvest 4	Jade	388.2d	4.27d	4.75a	1.18c	0.28bc
	Putland	812.4h	9.4g	–	2.22d	0.23b
	Berken	485.8e	4.48de	5.82b	0.82c	0.24b
	Celera II	576.6f	3.97d	6.28c	0.92c	0.23b

Mean values for each parameter and harvest date are accompanied by letters to indicate significant differences from the variety*harvest date interaction.

### Root System Architecture (RSA) and Root Morphology

**Figure 3:**
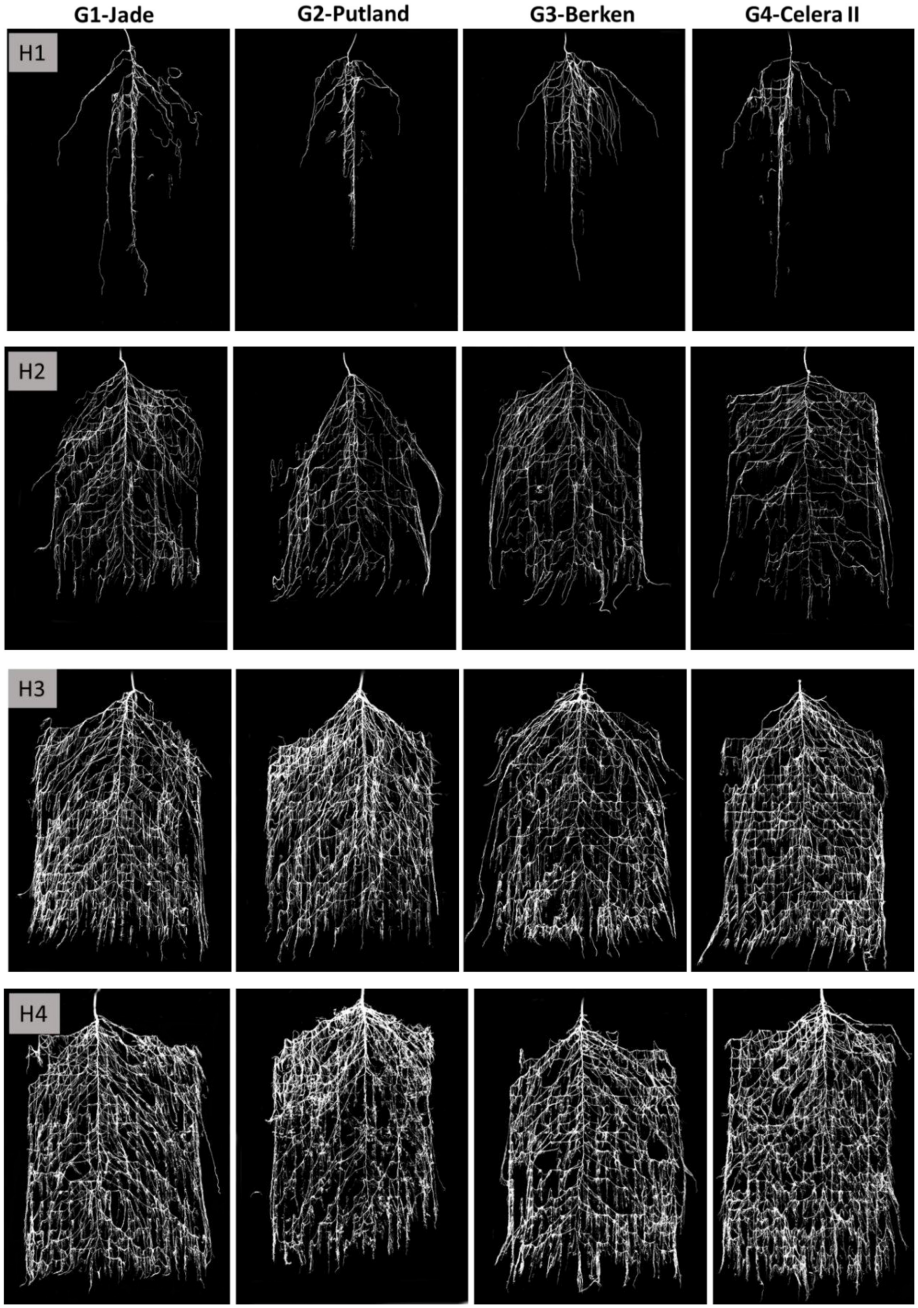
Intact root growth images on the pinboard (after processing with adobe photoshop) at four harvests (H1, H2, H3 and H4) for mungbean varieties Jade, Putland, Berken and Celera II. These images were analyzed with WinRhizo for key root traits measurements.

**Figure 4:**
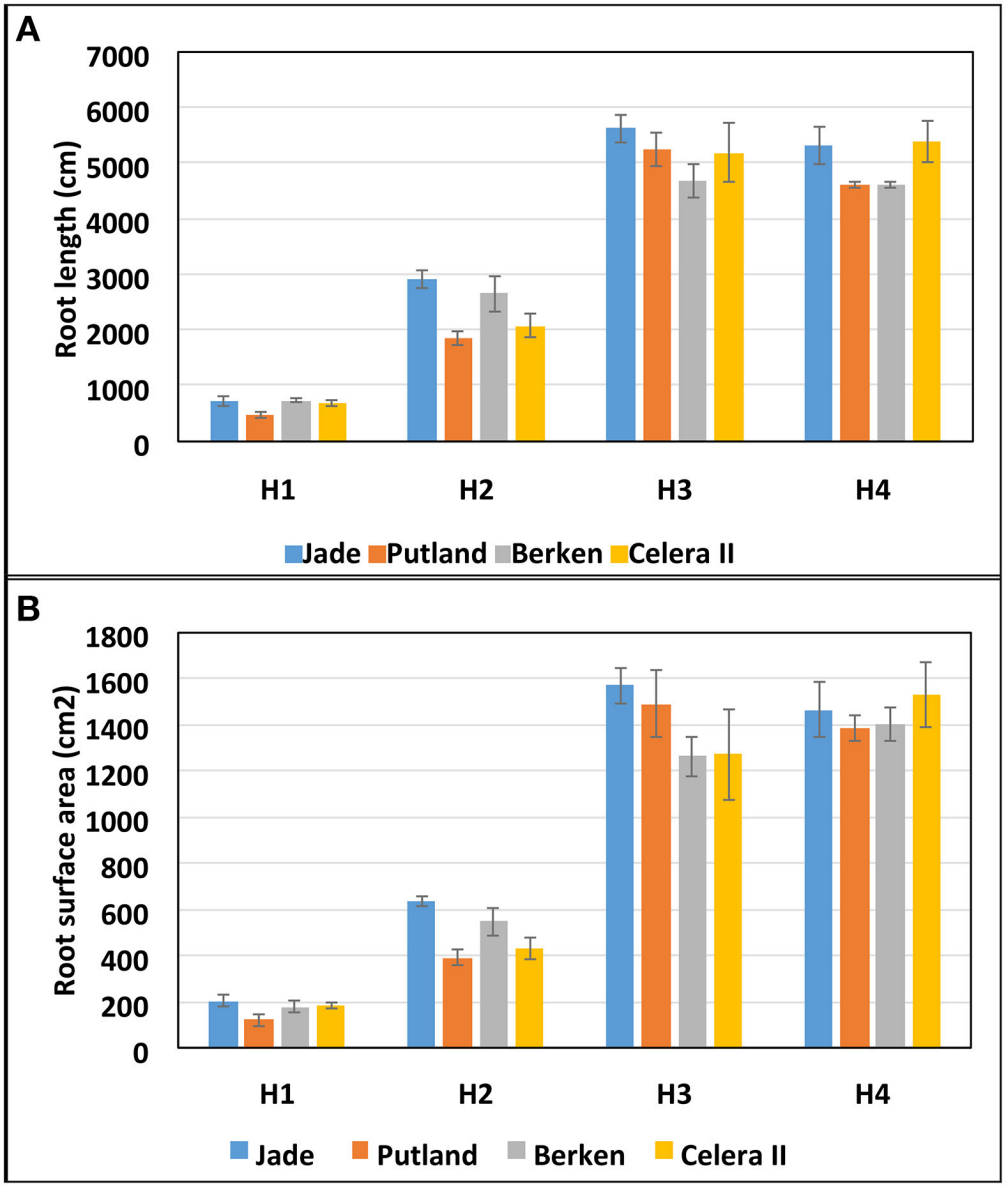
Root morphometric measurements **(A)** total root length and **(B)** root surface area at four harvests (H1, H2, H3, and H4) for four mungbean varieties (Jade, Putland, Berken and Celera II). Bars represent standard error of means.

Visual records of growth and development of intact root systems for the experiment duration are presented in the Figure 3, while root morphometric data (from manual and WinRhizo measurements) are presented in the Figure 4. At early growth stages (H1 and H2), the early maturing varieties Jade and Berken, and to some extent Celera II, showed more vigorous root growth and root branching deeper in the root chamber, whereas the late maturing Putland showed relatively slow root growth and development (Figures 3, 4). Visually, after 20 days of growth at H1 the tap root of the early maturing varieties had effectively reached the bottom of the chamber (i.e., 60 cm), whereas the late maturing Putland had only reached 50 cm deep at that time (Figure 3). However, in the later stages of growth all varieties showed similar tap root lengths of between 60 and 70 cm, with no significant differences between them (Figure 3). Early elongation and proliferation of the second and third order lateral roots was significantly more rapid for the early maturing varieties at H1 and H2 (Figure 3), but this trend was reversed in the mid and later growth stages (H3 and H4), when the later maturing Putland showed significantly more root growth. Visually, Putland and to some extent Celera II, grew relatively thicker roots in the surface soil, whereas Jade and Berken showed more root branching in the deeper layers (Figure 3). Jade recorded the greatest root surface area in the bottom layer, whereas Putland had the most root surface area in the top layers (see Supplementary Table 1). Jade also had greater root surface area in the bottom than the top 30 cm of the chambers, while Putland and to some extent Celera II showed an opposite trend. Berken had similar root surface areas in the top and bottom chamber (see Supplementary Table 1).

Key root morphometric measurements such as the total root length (Figure 4A) and root surface area (Figure 4B) supported the visual observations shown in the Figure 3, with the differences in root growth between varieties at different growth stages of particular interest. The early maturing varieties Jade and Berken, and to a lesser extent Celera II, showed greater root growth (length and surface area) early in the season (H1 and H2—Figures 4A,B) than Putland, with this trend reversed in later growth stages (H3 and H4).

Number of root tips, forks (indicating root branching patterns) and crossing (overlapping) were also quantified with WinRhizo. These root parameters were closely related to each other, so only the number of root tips have been presented (see Supplementary Table 1). Putland and Berken showed around 37% fewer root tips than Jade and Celera II. On average, the number of root tips increased *ca*. 11-fold (from 1,100 to 13,300) as plants aged. Specific root mass (root mass/root length) of the late maturing Putland was 20% to 50% greater than the three early maturing varieties, with Celera II and Berken showing lower specific root mass than Jade (Supplementary Table 1).

Root growth angle appeared to increase from H1 and H4 in all varieties, with Jade showing a lower root growth angle than the Celera II (see Supplementary Table 1). The number of nodules also increased over time in all varieties, ranging from 3 to 9 plant^−1^, but there were no significant varietal differences (see Supplementary Table 1).

### Development of Roots and Shoots

**Figure 5:**
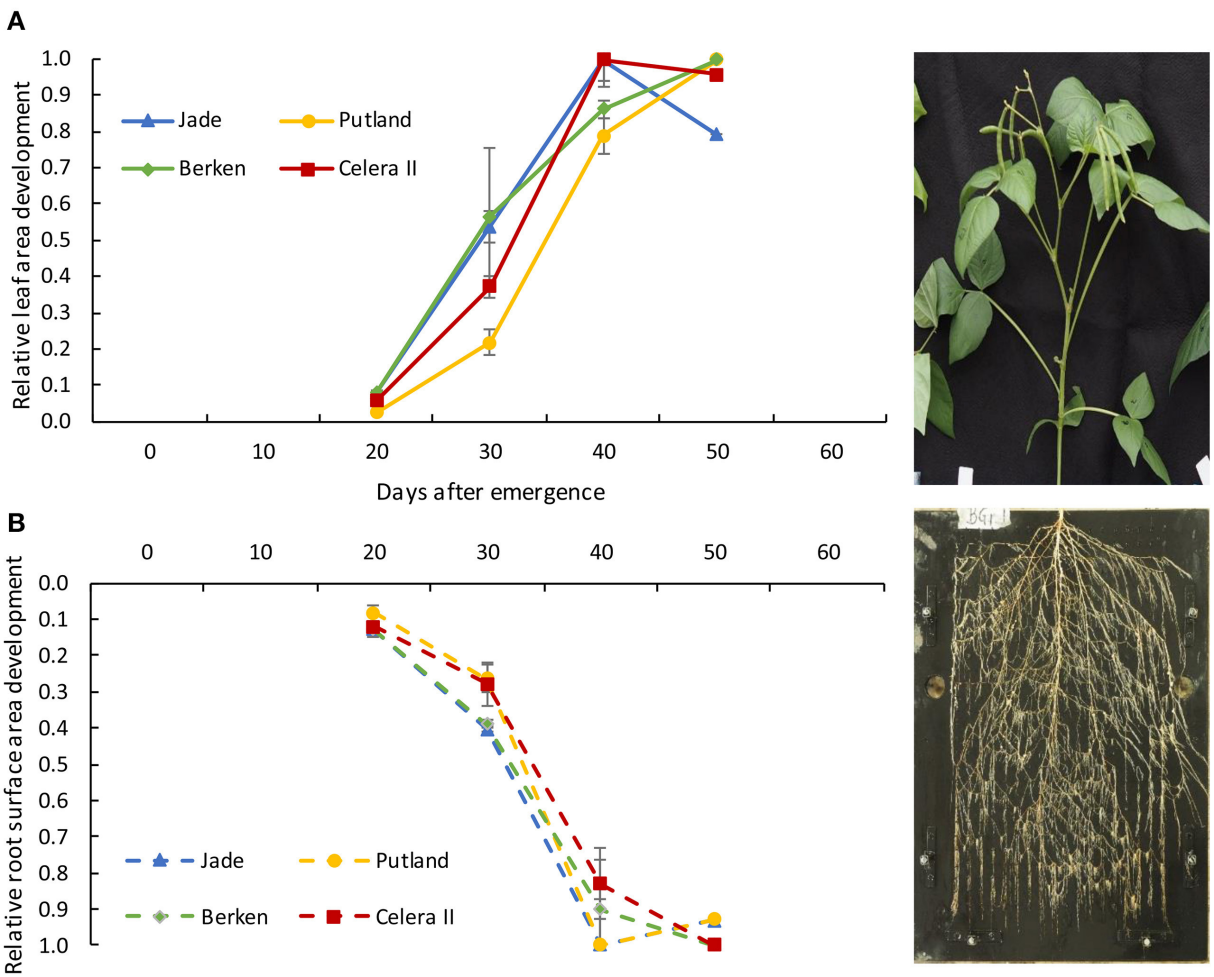
Relative development of leaf area **(A)** and root surface area **(B)** from 20 days to 50 days of plant growth for four varieties; Jade, Putland, Berken and Celera II. Error bars indicate +/- standard error of means. The photograph represents the figures for the directional growth of shoot **(A)** and root **(B)**.

**Figure 6:**
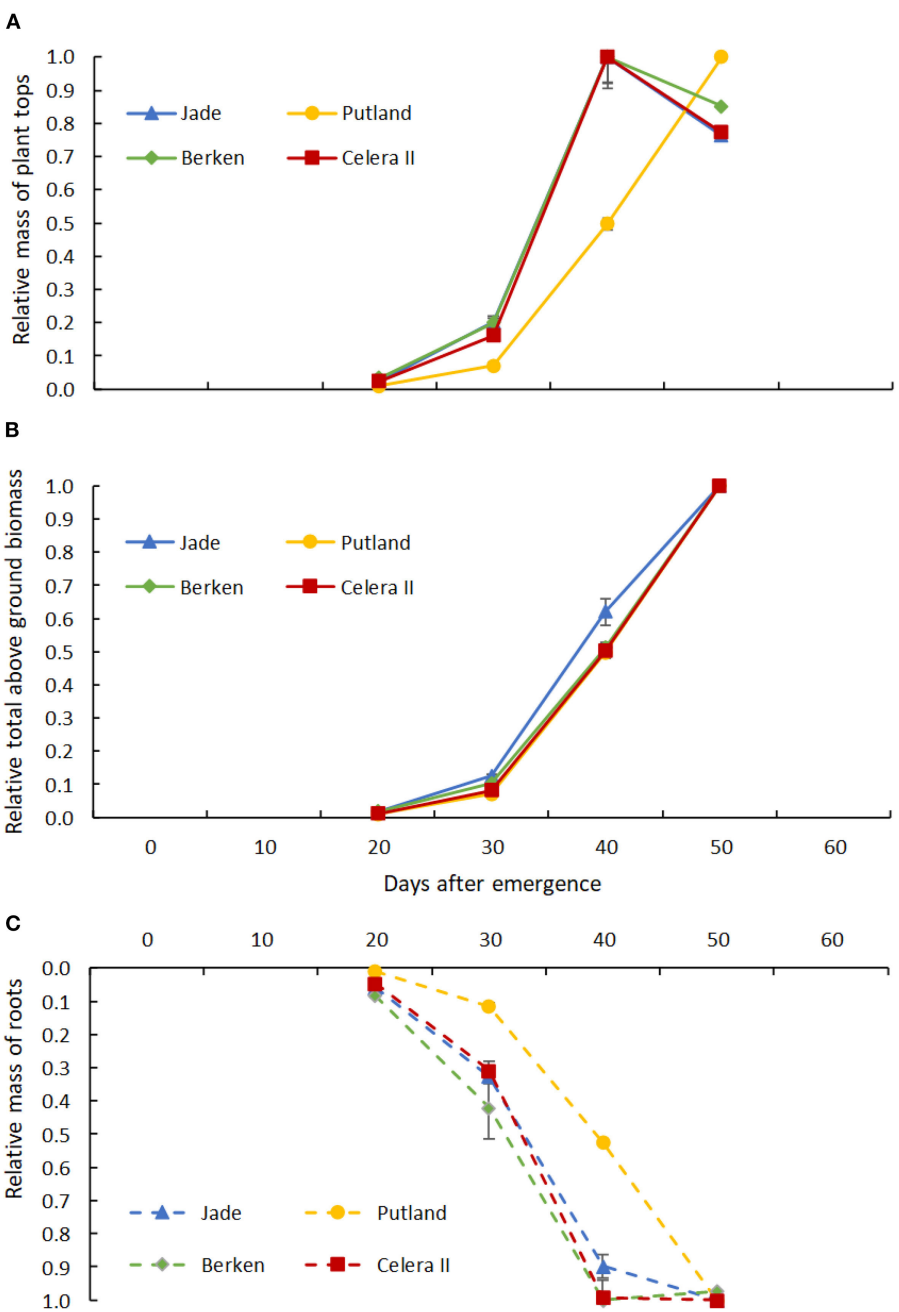
Relative mass of **(A)** plant tops (leaf + stem) and relative mass of **(B)** aboveground biomass (leaf + stem + pods) to relative mass of root **(C)** from 20 days to 50 days of plant growth for four varieties; Jade, Putland, Berken and Celera II. Error bars indicate +/− standard error of means.

Relationships between shoot and root growth parameters were constructed for individual varieties, because of contrasting growth patterns evident between early and late maturing varieties (Table 1; Figures 5, 6). Since the key root morphological parameters such as the total root length and root surface area were highly correlated with each other, and the root surface area (product of root length and root diameter) showed slightly better relationships with the shoot parameters, we used the development of root surface area (Figure 4B) to compare with the development of leaf surface area (Table 1). The relationship was curvilinear over time (H1 to H4) for all varieties, with root surface area increasing with leaf area until H3, after which root surface area did not increase further for any variety (data not presented). Leaf area also did not increase between H3 and H4, except for Putland. The relative development of both root surface area and leaf area are presented in the Figure 5, with values for each harvest expressed relative to the maximum value recorded for each parameter (shown in Supplementary Table 1).

A similar analysis was conducted for aboveground and belowground dry matter accumulation (Table 1), with the former considered as a whole (i.e., leaf, stem and pods where these were present) or simply as vegetative material (leaf and stem). Significant interactions occurred between varieties and growth stages for above ground shoot mass, but not for the belowground root mass (Table 1). During the early growth stages, shoot mass was higher for the early maturing varieties than the late maturing Putland, which was consistent with the more rapid development of leaf area (Figure 5). All three relatively early maturing varieties had greater shoot mass than the late maturing Putland at H3 (Table 1). However, between H3 and H4 Putland accumulated >80% more shoot mass compared with the other varieties. In contrast to Putland, while other varieties did not grow any more shoot mass, or shoot mass appeared to decrease slightly during this period, these varieties instead accumulated biomass in pods. When these results were considered in relative terms (Figure 6), all varieties showed similar patterns of total above ground dry matter accumulation but different patterns of below ground dry matter accumulation, with root DM no longer increasing in any of the earlier maturity lines beyond 40 DAE (Figure 6). The contrast between total above ground dry matter and vegetative dry matter production is illustrated in Figure 6, with the latter showing that accumulation of vegetative dry matter and root dry matter were tightly correlated in all varieties.

## Discussion

The primary aim of this study was to observe and understand the dynamic nature of root growth and root system architecture (RSA) and its relationship with shoot growth (vegetative and reproductive) over time for mungbean varieties differing in maturity times. In this study, the homogenous soil profiles in the root chambers, adequate supplies of water and nutrients and semi-controlled experimental conditions allowed varietal differences in patterns of root and shoot growth to be expressed without the confounding biotic and abiotic effects commonly encountered in field studies.

### Phenological Effects on Root Growth Dynamics and RSA

Differences in RSA between the late maturing variety Putland and the early maturing varieties Jade, Berken and Celera II were significant, and also differed between growth stages. The early maturing varieties showed more rapid root growth, developing denser and more prolific root systems at greater soil depths earlier in the crop life cycle (i.e., H1 and H2—Figure 3). In contrast, the later maturing Putland developed greater root length and relatively thicker roots in the surface soil during the later growth stages (i.e., H3 and H4). The observed visual differences were consistent with the image analysis of intact RSA and morphometric measurements made using WinRhizo PRO. Similar findings have also been recorded for sorghum (Singh et al., 2012) and rice (Uga et al., 2013), with early maturing varieties exhibiting relatively enhanced root growth and development in deeper soil layers during early growth stages compared to later maturing varieties. These studies also found that the late maturing lines had greater root lengths and thicker roots in the topsoil than in the deeper soil layers. It could be hypothesized that the root thickening and an increase in the root surface area for the late maturing variety was an artifact of growing in a restricted soil volume, and that root length and surface area would have continued to increase in a larger pot. However, root thickening and an increase in root surface area in Putland occurred only in the surface soil (Figure 3 H3–H4; Supplementary Table 1), whereas early maturing varieties indicated relatively greater root surface area in the bottom section of the soil profile (for example, Jade, Supplementary Table 1). This suggests that the observed thickening and increase in the root surface area in the surface soil by Putland may not be due to restricted soil volume, and this is further supported by the observation of continuous and statistically significant increases in the number of root tips from H1 to H4 (Supplementary Table 1).

Root length, root surface area, number of root tips, root collar diameter and specific root mass also changed over time for each variety (Table 1; Supplementary Table 1). Growth of these parameters differed between early and late growth stages, reflecting the dynamic nature of root growth and RSA in mungbean. Chen et al. (2017) also reported root vertical angles changing with depth while phenotyping the dynamics of wheat (*Triticum. aestivum*) RSA in the field over time. Similarly, Hund et al. (2009) reported a linear trend in the axial root length at the early growth stages or seedling stage for maize (*Zea mays*), whereas Barraclough and Leigh (1984) reported a curvilinear trend in the root growth pattern until flowering for wheat. The dynamic nature of root system development observed in our studies was linked to changes in the shoot and reproductive growth observed over the course of this experiment (as discussed later).

Early maturing varieties showed greater total root length and root mass at early growth stages (Figure 4; Table 1). However, differences in the total root length were less pronounced at the mid to late growth stages compared with that of Putland, which recorded large increases in the root mass from H3 to H4. Variable growth of these two key root morphological traits (root mass and length) resulted in a lower specific root mass (mass/length) for the early maturing varieties than the late maturing variety (see Supplementary Table 1). Lower root mass per unit length suggested a lower carbon requirement for root construction in the early maturing varieties compared to Putland, and the lighter roots in the early maturing types was also reflected in a significantly lower root mass compared with Putland at H4 (Table 1). The lighter roots of early maturing types have been identified as a preferred ideotype for root systems that have potentially reduced construction and maintenance costs in other studies (Lenochova et al., 2009; Zhu et al., 2010; Lynch, 2013). The root systems in these early maturing mungbean varieties, therefore, were not only characterized by the establishment of a deeper root system earlier in the growing season, with the potential to extract more water from deeper profile layers, but were able to achieve this at a lower C cost.

Wasson et al. (2012) advocated for greater root length densities at depth and reduced density in the topsoil to favor deep soil water extraction, and so the early maturing mungbean varieties in this study would potentially seem well-adapted to such conditions—at least during early growth stages. Conversely, the relatively thicker and more prolific surface root development in the later maturing Putland could provide better anchorage to support the vigorous shoot growth and larger biomass that accumulated during mid to late growth stages, and would be more effective in utilizing smaller rainfall events that wet only the top soil layers. Such root systems may also have a better horizontal spread and greater water extraction at a distance from the plant row, as observed for shallow rooting sorghum (*Sorghum bicolor*) lines (Singh et al., 2012). Greater nutrient acquisition has also been associated with increased soil exploration by roots in surface layers, especially in the case of immobile nutrients such as phosphorus (Bonser et al., 1996; Lynch and Brown, 2001). Whilst our study is not able to address the potential for improved nutrient foraging by the greater allocation of root biomass to surface soil layers with Putland, it is important to note that the increased root mass was not accompanied by an equivalent increase in root surface area (Figure 4B) in that part of the profile. This would be an important factor influencing the efficiency of recovery of nutrients like phosphorus, where diffusive supply over short distances is a key factor determining nutrient acquisition. Singh et al. (2012) noted that shallower rooting sorghum genotypes with wider root growth angle and thicker roots in the surface soil extracted more water from the surface layer in a drying soil. Shallower root systems are also more adapted to relatively shallower soil profiles and wider row spacing configurations (Singh et al., 2012). However, under terminal drought conditions shallow rooting varieties like Putland may underperform compared with deeper rooting early maturing varieties (Jade, Berken and Celera II), which would appear to be more suited to deeper soil profiles and narrow row spacing configurations.

The early maturing variety Jade showed relatively narrower root growth angle in this study. Root growth angle has been noted to be the key indicator of a deep or shallow rooting genotype (Singh et al., 2011; Uga et al., 2013; Chen et al., 2017) and plays a major role in determining RSA. However, while root growth angle is primarily governed by plagiogravitropism (Nakamoto, 1994), it can also be influenced by other factors such as soil strength and soil water, (Nakamoto, 1993, 1994; Trachsel et al., 2011) soil temperature (Tardieu and Pellerin, 1991) and soil nutrition, especially phosphorus. Evidence from different crops has indicated that genotypes with narrow root growth angles are not only deep rooting, but they also tend to grow and develop more rapidly in both above and below-ground components, leading to early flowering and maturity. This characteristic was initially noted for genotypes of rice (Uga et al., 2013) and sorghum (Singh et al., 2012). The root morphological traits such as deeper and more prolific root development that were observed in early maturing mungbean varieties in this study have been linked to adaptation to water-limited environments in sorghum (Ludlow et al., 1990; Tsuji et al., 2005; Singh et al., 2012), rice (*Oryza sativa*) (Ekanayake et al., 1985; Kato et al., 2006; Uga et al., 2013), and maize (Hund et al., 2008).

### Interactions Between Phenological Development and Plant Growth in Mungbean Varieties

The early maturing varieties Jade and Berken showed more rapid dry matter accumulation in both tops and roots during the early growth stages (i.e. H1 and H2—Table 1), slightly faster than Celera II and significantly more than Putland, with these differences consistent with more rapid leaf area development during that time (Table 1). To support this vigorous root and shoot growth, early maturing varieties also showed a greater dry matter production per unit leaf area, indicative of an increase in efficiency of resource capture or use efficiency.

Differences in biomass production between varieties had largely disappeared by H3 and were not evident at all at H4—even though the dry matter constituents now differed between varieties due to the addition of pods in Jade, Berken and Celera II (Table 1). The relative accumulation of above and below ground dry matter was strongly correlated in Putland, which exhibited solely vegetative growth during the study, but not in the earlier maturing varieties in which accumulation of root dry matter had effectively ceased after H3 (40DAE—Figure 6A). The contrast between relative accumulation of total and vegetative above ground dry matter (Figure 6) illustrated the significant impact of commencement of pod development on growth of other plant parts, both above and below ground, in the earlier maturing varieties. Pod establishment from 40 DAE resulted in the complete cessation of vegetative and root growth in the early maturing varieties, while growth of both components continued unabated in Putland. The photoperiod sensitivity of the variety Putland may have contributed to the extended vegetative phase in this study, as daylengths >13 h would likely have contributed to delays in the onset of flowering and subsequent reproductive development”. Nevertheless, major changes in assimilate distribution patterns were triggered by the early establishment of the pods as sinks for assimilate, with these changes having significant implications for both the ability to efficiently exploit stored soil water deeper in a soil profile, and for optimum planting configurations (row spacings and plant densities) in varieties with differing phenology. Both Nord and Lynch (2009) and Lynch (2013) have previously highlighted that the transition from vegetative to reproductive growth has important implications for soil resource acquisition.

### The Relationship Between Root and Shoot Growth Dynamics

Rapid expansion of leaf and root surface areas are critical to the establishment of structural frameworks needed to capture resources for subsequent crop growth (Figures 5, 6), with the strong positive correlation between these indicators of above and below-ground resource capture noted in many other species. For example, Grieder et al. (2014) noted positive relationships between leaf area and root length and rooting depth for maize genotypes. The vigorous root growth and more rapid rates of root extension seen in the early maturing mungbean varieties in our study are consistent with acquisition of sufficient water and nutrients to be able to support the rapid shoot growth observed in these varieties. This synchrony of resource capture is essential to the rapid development of a competitive crop canopy consistent with the rapid phenological advancement during shorter growing seasons.

Poorter et al. (2009) reported close relationships between the rate of photosynthesis and shoot growth, and as noted by Lynch (2013), shoot characteristics that enhance the conversion of water or nitrogen to carbon and energy in photosynthesis will also permit greater root growth, and hence greater soil resource acquisition. This was illustrated for sunflower by Aguirrezabal and Tardieu (1996), who reported that root extension rate was related to photosynthetic photon flux density and leaf area development. An improved carbon assimilation rate was able to support increased rates of root elongation, increased root branching and overall greater root length (Aguirrezabal et al., 1993), subsequently increasing the water and nutrient uptake. While the rate of photosynthesis was not determined in our mungbean study, the greater dry matter production per unit leaf area during early growth stages was consistent with higher photosynthetic rates in the early maturing mungbean varieties. Arai-Sanoh et al. (2014) suggested that a high flux of cytokinins, mostly synthesized in the roots and root tips, could contribute to the high photosynthetic rate. Two of the three early maturing varieties in our study showed greater number of root tips (Supplementary Table 1).

The relationship between root growth and reproductive growth and/or yield parameters is complex, as both are influenced by biotic and abiotic factors. Watt et al. (2013) were able to relate a rapid seedling root screen with the rooting depth in vegetative growth stages, but not for the reproductive growth stage. Our mungbean study did observe that increased rates of deeper root development during early growth stages were correlated with early flowering and podding characteristics in the varieties studied. Conversely, the early onset of reproductive growth and pod establishment had very strong impacts on partitioning of carbohydrate to root growth and root dry weight during later growth stages. This would result in an erosion of the benefit of rapid root establishment in the earlier maturing varieties, given that later maturing lines like Putland could continue to establish more roots over an extended vegetative period. The relative benefits of these contrasting phenologies and patterns of root development will differ between production environments and will also likely impact on the optimum agronomic management strategies.

## Conclusions

Knowledge of the relationship between various root traits and plant productivity is necessary to effectively target management and breeding strategies to improve crop productivity. Our studies indicated that early maturing mungbean varieties were characterized by a combination of traits that contributed to more vigorous root and shoot growth during early growth stages than in a later maturing variety. Traits such as the deeper, longer and lighter roots found in these varieties would be expected to confer better adaptation to water-limited environments, although the rapid onset of reproductive growth and the cessation of subsequent root growth may limit the impact of these traits in the field. In contrast, the later maturing variety exhibited relatively thicker roots in the topsoil layers that could provide a better anchorage to support the larger plants with presumably a greater abundance of maturing pods that an extended growing season would facilitate, in the absence of other constraints.

While this study was conducted under conditions of adequate water and nutrient supply, the differences in observed root traits between varieties would suggest differential adaption to environments where water and/or nutrients may be suboptimal during the growing season. Mungbean crops are generally grown in marginal environments with limited soil moisture, and late maturing varieties with limited “in-crop” seasonal rainfall may have to survive on stored moisture in deeper layer of soil. However, in the wet seasons, crops may not use much of the deeper profile moisture at all, therefore, the type of the root system that will provide a water advantage will be entirely dependent on the growing seasons targeted and soil depth. Although our findings are based on a limited number of genotypes and there is a need for broader examination of variation, but we hypothesize that root systems that developed on the late maturing variety may be better adapted to relatively shallower soil depths, surface stratified nutrient reserves and wider row spacing configurations, whereas the narrower and deeper rooting observed in the early maturing varieties may be more suited to deeper soil profiles with more uniform nutrient distributions and narrow row configurations.

## Data Availability Statement

The original contributions presented in the study are included in the article/Supplementary Material, further inquiries can be directed to the corresponding author.

## Author Contributions

VS contributed to the conceptualization of the research, experimental set up, data collection, result analysis, and writing of the main manuscript text. MB contributed to the conceptualization and improved the text, and also led the project. Both authors contributed to the article and approved the submitted version.

## Conflict of Interest

The authors declare that the research was conducted in the absence of any commercial or financial relationships that could be construed as a potential conflict of interest.

## Publisher's Note

All claims expressed in this article are solely those of the authors and do not necessarily represent those of their affiliated organizations, or those of the publisher, the editors and the reviewers. Any product that may be evaluated in this article, or claim that may be made by its manufacturer, is not guaranteed or endorsed by the publisher.
