# Supplementary material for: Genotypic Variability in Architectural Development of Mungbean (Vigna radiata L.) Root Systems and Physiological Relationships With Shoot Growth Dynamics
Source: Front Plant Sci. 2021 Aug 19;12:725915. doi: 10.3389/fpls.2021.725915 (PMC8417475; doi:10.3389/fpls.2021.725915)
Supplement: Supplementary file 1 [file Table_1.docx]

# **SUPPLEMENTARY TABLE 1**: Main treatments (genotype and harvest) and interactions (GxH) effects (*P*) on various plant parameters with their means and least significant differences (lsd at 5% probability) of means.

|  | **Genotype (G)** | | | | | | **Harvest (H)** | | | | | | **GxH** | |
| --- | --- | --- | --- | --- | --- | --- | --- | --- | --- | --- | --- | --- | --- | --- |
| **Parameters** | Jade | Putland | Berken | Celera II | *P* | lsd (0.05) | H1 | H2 | H3 | H4 | *P* | lsd (0.05) | *P* | lsd (0.05) |
| Plant height (cm) | 30.5 | 25.3 | 29.0 | 31.3 | 0.002 | 1.75 | 9.0 | 23.5 | 42.2 | 40.8 | <0.001 | 2.20 | <0.001 | 3.50 |
| No of branches | 6.1 | 7.0 | 5.9 | 7.2 | <0.001 | 0.42 |  | 3.3 | 7.2 | 8.8 | <0.001 | 1.24 | <0.001 | 0.84 |
| Total number of leaves | 12.6 | 17.6 | 12.4 | 14.8 | <0.001 | 1.17 | 5.0 | 13.5 | 18.0 | 20.9 | <0.001 | 2.01 | <0.001 | 2.35 |
| Fully expanded leaf | 9.5 | 14.1 | 10.5 | 12.6 | <0.001 | 0.31 | 2.0 | 11.0 | 15.9 | 21.1 | <0.001 | 0.45 | <0.001 | 0.63 |
| Total leaf Area per plant (cm^2^) | 316 | 364 | 310 | 357 | <0.001 | 23.8 | 31 | 227 | 528 | 553 | <0.001 | 35.2 | <0.001 | 47.6 |
| Individual leaf size (cm^2^) | 22.5 | 15.9 | 22.0 | 20.3 | <0.001 | 1.47 | 6.3 | 16.8 | 29.6 | 27.6 | <0.001 | 2.12 | <0.001 | 2.95 |
| Shoot dry wt (g) | 3.1 | 3.2 | 2.9 | 2.4 | <0.001 | 0.25 | 0.1 | 0.9 | 5.2 | 5.3 | <0.001 | 0.60 | <0.001 | 0.49 |
| Root angle (degree) | 43.7 | 46.0 | 46.1 | 48.3 | 0.017 | 3.03 | 42.6 | 43.6 | 46.4 | 49.4 | 0.014 | 4.19 | 0.132 | 6.05 |
| Root collar diameter (mm) | 3.4 | 3.4 | 3.0 | 3.1 | <0.001 | 0.18 | 1.6 | 2.6 | 4.2 | 4.5 | <0.001 | 0.19 | <0.001 | 0.35 |
| Root dry wt (g) | 0.74 | 0.80 | 0.54 | 0.54 | <0.001 | 0.07 | 0.05 | 0.31 | 1.01 | 1.25 | <0.001 | 0.097 | <0.001 | 0.142 |
| Number of nodule | 6.9 | 4.0 | 7.0 | 6.3 | NS | - | - | 2.6 | 6.8 | 7.9 | 0.021 | 3.91 | NS | - |
| No of pods | 6.3 | 0.0 | 7.5 | 13.8 | <0.001 | 1.19 | - | - | 5.0 | 9.5 | - | - | - | - |
| Pod weight (g) | 4.7 | - | 5.8 | 6.3 | <0.001 | 0.09 | - | - | - | 5.6 | - | - | - | - |
| Biomass (g) | 5.2 | 4.0 | 5.0 | 5.0 | NS | - | 0.2 | 1.2 | 6.2 | 11.0 | <0.001 | 0.70 | 0.003 | 0.58 |
| Root:Shoot Ratio | 0.32 | 0.27 | 0.27 | 0.28 | 0.099 | 0.028 | 0.35 | 0.36 | 0.20 | 0.23 | <0.001 | 0.014 | 0.021 | 0.045 |
| DM/Leaf area (g/cm^2^) | 0.014 | 0.008 | 0.013 | 0.010 | <0.001 | 0.0006 | 0.005 | 0.005 | 0.012 | 0.021 | <0.001 | 0.0012 | <0.001 | 0.0012 |
| Root Length(cm)_WinRh | 3900 | 2769 | 3075 | 3334 | 0.007 | 330.6 | 626 | 2300 | 5218 | 5058 | <0.001 | 400.9 | 0.154 | 661.1 |
| Root Diam(mm)_WinRhi | 0.85 | 0.83 | 0.82 | 0.80 | 0.083 | 0.048 | 0.85 | 0.67 | 0.86 | 0.92 | <0.001 | 0.068 | 0.125 | 0.096 |
| RootSurfArea(cm^2^) | 1046 | 761 | 824 | 872 | 0.155 | 1226.0 | 168 | 487 | 1423 | 1455 | <0.001 | 1386.0 | NS | - |
| RootSurfArea_Top (cm^2^) | 690 | 872 | 616 | 621 | 0.018 | 151.7 | - | - | - | - | - | - | - | - |
| RootSurfArea_Bottom (cm^2^) | 813 | 550 | 620 | 487 | 0.018 | 175.8 | - | - | - | - | - | - | - | - |
| Number of RootTips | 9198 | 6672 | 6747 | 9107 | <0.001 | 308.6 | 1170 | 6747 | 11434 | 13345 | <0.001 | 583.4 | 0.183 | 617.1 |
| Specific Root Mass*1000 (g/cm) | 0.17 | 0.20 | 0.15 | 0.13 | <0.001 | 0.007 | 0.07 | 0.14 | 0.19 | 0.26 | <0.001 | 0.007 | <0.001 | 0.013 |
